# Sexual Dimorphism in Bite Performance Drives Morphological Variation in Chameleons

**DOI:** 10.1371/journal.pone.0086846

**Published:** 2014-01-27

**Authors:** Jessica M. da Silva, Anthony Herrel, G. John Measey, Krystal A. Tolley

**Affiliations:** 1 Applied Biodiversity Research Division, South African National Biodiversity Institute, Cape Town, Western Cape Province, South Africa; 2 Department of Conservation Ecology and Entomology, Stellenbosch University, Stellenbosch, Western Cape Province, South Africa; 3 Département d’Ecologie et de Gestion de la Biodiversité, Centre National de la Recherche Scientifique/Muséum National d’Histoire Naturelle, Paris, Île-de-France, France; 4 Evolutionary Morphology of Vertebrates Research Group, Department of Biology, Ghent University, Ghent, East Flanders, Belgium; 5 Department of Zoology, Nelson Mandela Metropolitan University, Port Elizabeth, Eastern Cape Province, South Africa; 6 Department of Botany and Zoology, Stellenbosch University, Stellenbosch, Western Cape Province, South Africa; University of Sao Paulo, Brazil

## Abstract

Phenotypic performance in different environments is central to understanding the evolutionary and ecological processes that drive adaptive divergence and, ultimately, speciation. Because habitat structure can affect an animal’s foraging behaviour, anti-predator defences, and communication behaviour, it can influence both natural and sexual selection pressures. These selective pressures, in turn, act upon morphological traits to maximize an animal’s performance. For performance traits involved in both social and ecological activities, such as bite force, natural and sexual selection often interact in complex ways, providing an opportunity to understand the adaptive significance of morphological variation with respect to habitat. Dwarf chameleons within the *Bradypodion melanocephalum-Bradypodion thamnobates* species complex have multiple phenotypic forms, each with a specific head morphology that could reflect its use of either open- or closed-canopy habitats. To determine whether these morphological differences represent adaptations to their habitats, we tested for differences in both absolute and relative bite performance. Only absolute differences were found between forms, with the closed-canopy forms biting harder than their open-canopy counterparts. In contrast, sexual dimorphism was found for both absolute and relative bite force, but the relative differences were limited to the closed-canopy forms. These results indicate that both natural and sexual selection are acting within both habitat types, but to varying degrees. Sexual selection seems to be the predominant force within the closed-canopy habitats, which are more protected from aerial predators, enabling chameleons to invest more in ornamentation for communication. In contrast, natural selection is likely to be the predominant force in the open-canopy habitats, inhibiting the development of conspicuous secondary sexual characteristics and, ultimately, enforcing their overall diminutive body size and constraining performance.

## Introduction

Evolutionary and ecological processes that drive adaptive divergence and, ultimately, speciation can be influenced by phenotypic performance in different environments. As new environmental niches become available for populations to exploit, morphological and physiological adaptations arise, often resulting in enhanced performance in the novel habitat [1]. Evidence for these adaptations can be found in the improved performance of animals in their new environment [1]. For example, habitat structure or complexity is known to influence a range of lizard behaviours, including communication and anti-predator defences. Densely vegetated, structurally complex habitats may afford lizards greater cover from avian predators. If indeed predation pressure is released in dense vegetation, chameleons may invest more in conspicuous features, such as ornamentation and bright colouration, for increased detectability to conspecifics. However, in less vegetated habitats, where visibility to predators is high, rather than being visible chameleons may need to be cryptic to avoid detection (e.g., [2], [3]). Because the head is involved in many ecologically and socially relevant activities, such as feeding, mating and aggressive interactions, its morphology and association to bite performance and habitat have been widely investigated to better understand the adaptive significance and the underlying processes shaping phenotypic variation within and between species (e.g., [4]–[15]). Many of these studies have shown that bite force is influenced by both natural and sexual selection, yet the relative contribution of these selective pressures remains difficult to unravel as they often interact in complex ways. Moreover, sexual and natural selection can act in opposite ways, with sexual selection favouring conspicuous coloration or ornamentation for effective communication and conflict avoidance, and natural selection favouring cryptic coloration and reduced ornamentation to avoid injury from predation, as well as high bite forces in the context of intraspecific encounters [16], [17]. This results in a trade-off between the two selective pressures, with the relative strength of natural and sexual selection on particular head traits being partly dependent on the environment (e.g., [18]–[20]). This complex interaction often results in interspecific variation; however, it can also lead to intraspecific variation in the form of varying degrees of sexual dimorphism (e.g., [21]–[24]), both of which have been shown to contribute significantly to adaptive radiations [1], [24].

**Figure 1:**
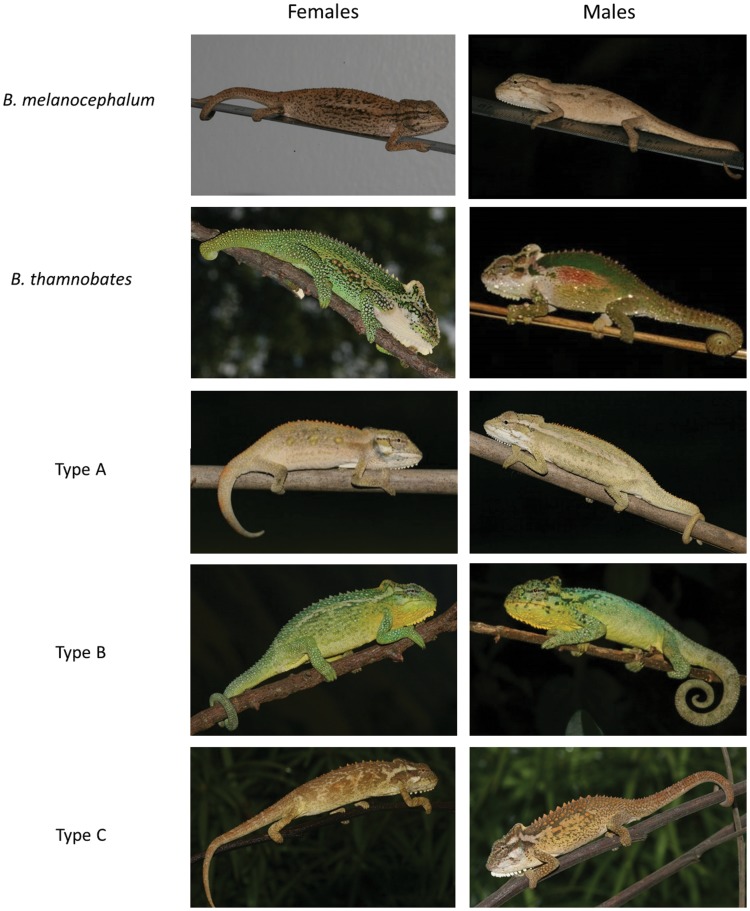
Photographs of the five dwarf chameleon forms within the *B. melanocephalum*-*B. thamnobates* species complex from southern KwaZulu-Natal Province, South Africa. Figure reprinted from [33].

Chameleons have radiated into multiple habitats, including forests, grasslands, heathlands, savannah, and desert; and their colonisation of these different niches corresponds with the emergence of these biomes on the landscape [25]. Indeed, chameleon morphology may be under rapid directional selection in instances where novel habitats are colonised [26]–[28], and this process may be well illustrated by a radiation of dwarf chameleons (*Bradypodion*) from KwaZulu-Natal (KZN) Province, South Africa. The species complex is comprised of five phenotypic forms, two of which are described species (*Bradypodion melanocephalum*, *Bradypodion thamnobates*) and the remaining three (Types A, B and C) designated as morphotypes [29]–[33] (Fig. 1). These forms have been defined based on their morphology, particularly in terms of ecologically relevant morphological traits [33]. All forms are allopatric in distribution, but mitochondrial markers show they lack the divergence expected at the species level, which reflects the recent nature of the radiation [27], [28]. This lack of genetic differentiation has led some to deduce that the complex is comprised of phenotypically plastic forms of a single species. However, this hypothesis is unlikely, given that common garden experiments showed that *B. melanocephalum* and *B. thamnobates* juveniles developed the phenotype of their parent populations, regardless of the habitat in which they were raised [34]. At present, it is likely that no gene flow takes place between forms given that the habitats in which they occur are fragmented and isolated. There are also ecological differences between their macro- and micro-habitats, with *B. melanocephalum* and Type A occupying more open-canopy habitats (e.g., grasslands), which contain densely clustered, vertically-oriented vegetation for chameleons to perch upon; while *B. thamnobates* and Types B and C occupy closed-canopy habitats (e.g., forests, transformed landscapes) that contain broader perching substrates arranged both vertically and horizontally [33]. These ecological differences were found to correlate to functional differences in forefoot grip strength, suggesting that the forms are adapted morphologically to their different environments [35]. However, variation in head size and shape was found to be the most important component in differentiating between phenotypic forms in this radiation, accounting for approximately half of the total variation in both sexes [33]. Moreover, the degree of sexual dimorphism varied between forms, with little to no dimorphism in head size and shape detected among open-canopy habitat chameleons, yet extensive dimorphism among the closed-canopy *B. thamnobates* [33]. As such, it is expected that considerable sexual and interspecific (interform) variation will be uncovered in bite performance, lending further support for the designation of this radiation as adaptive.

Like most lizards, dwarf chameleons use their heads in intraspecific communication signalling to rivals that confrontations can be harmful, and displaying to females to assess their willingness to mate [29], [30], [36]–[40]. Considering that different structural habitats can select for different types of communication behaviour [2], [3], [41], [42], the effectiveness of a particular head design may depend upon the environment. Given that closed-canopy habitats can constrain the effectiveness of aerial predators, sexual selection may be the predominant force within these habitats, enabling chameleons, especially males, to invest more in ornamentation, such as the casque, for communication; while, in the open-canopy habitats, natural selection may outweigh sexual selection to increase crypsis [22], [36], [43]. This likely explains why chameleons with large heads and ornaments (*B. thamnobates* and Types B and C) occupy closed-canopy habitats, while those with proportionally smaller heads and ornaments (*B. melanocephalum* and Type A) typically occupy more open-canopy habitats [29], [33]. If differential degrees of natural and sexual selection are, in fact, influencing chameleon head morphology between and within these habitats, this should be reflected in their bite performance and in the morphological features used to produce it. Accordingly, if ornaments are honest signals, in closed-canopy habitats, bite force should correlate best to ornamentation, especially in males. The result would be high levels of sexual dimorphism, with males generating a greater force to assist them during intrasexual competitions. In contrast, in open-canopy habitats, bite force is expected to correlate with non-ornamented, functional characters, with the proportionally larger headed chameleons producing a greater force, irrespective of sex. Regardless of the degree of natural selection within each habitat, its influence between habitats is expected to be strong enough to ensure that larger headed chameleons possess a harder bite, which in this radiation would be the closed-canopy forms.

To test these predictions and gain insight into the adaptive nature of the chameleon head within this recent radiation, we use a combination of morphometric and bite force data for multiple phenotypic forms. Specifically, we investigate whether the heads of the phenotypic forms and sexes are morphologically and functionally differentiated with respect to habitat structure, and which morphological variables are most closely associated with bite force within each form. The latter allows for inferences to be made regarding ornamental features and behaviour.

## Materials and Methods

### Ethics Statement

Ethics clearance was obtained from Stellenbosch University (Clearance No. 2009B01007) and the South African National Biodiversity Research (Clearance no. 0010/08), and permits for scientific research and collections were obtained from Ezemvelo KZN Wildlife (OP 3538/2009; OP 4351/2009; OP 4596/2010), permitting the collection and handling of the lizards.

### Study Sites and Sampling Procedure

**Figure 2:**
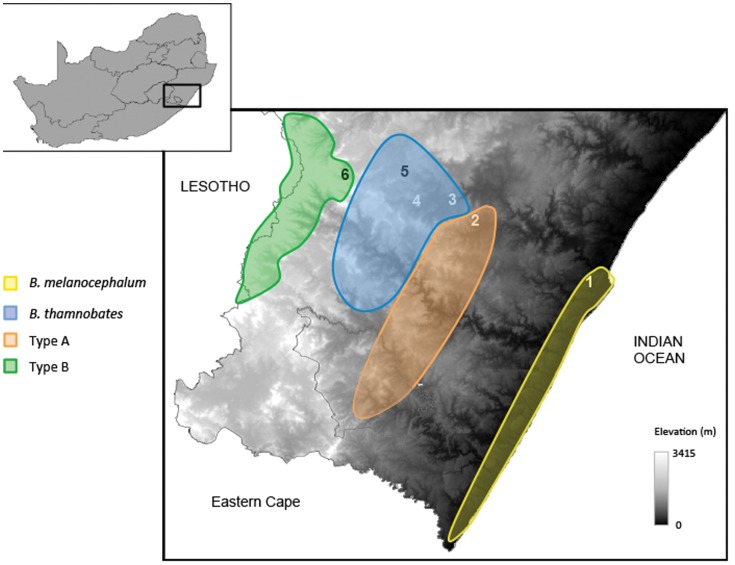
Distributions of four of the five phenotypic forms of the *B. melanocephalum*-*B. thamnobates* species complex. Numbers indicate field sites sampled in this study: 1, Durban; 2, Hilton; 3, Karkloof; 4, Howick; 5, Dargle; 6, Nottingham Road; 7, Kamberg Nature Reserve.

A total of 155 dwarf chameleons (79 males; 76 females) representing four of the five phenotypic forms of the *B. melanocephalum-B. thamnobates* species complex were sampled from six field sites within southern KZN (Fig. 2) between January and February 2010. These animals are a subset of individuals sampled for a previous ecomorphological study [33]. Although sampled, Type C was not included due to insufficient sample sizes. Animals were collected at night and geo-referenced at the exact location each chameleon was found. They were placed in separate cloth bags then brought back to the field base overnight, where they were measured and their bite force tested the subsequent day. Once all data were collected, animals were released at the exact site of capture.

### Morphometrics

**Figure 3:**
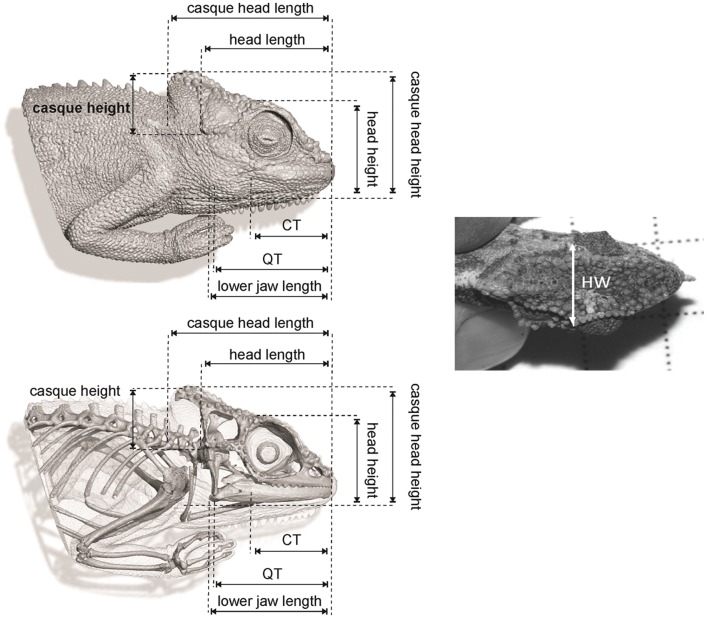
Nine head measurements recorded for each chameleon. Images on the left are based on a µCT-scan, courtesy of R. Boistel, Université de Poitiers. CT, coronoid process of mandible to snout tip; QT, posterior surface of quadrate to snout tip; HW, head width.

For all chameleons, snout-vent length (SVL) and nine head measurements (ornamented or non-ornamented) were measured to the nearest 0.01 mm using digital callipers (Table 1, Fig. 3). The non-ornamented measurements included lower jaw length (LJL), head length (HL), head width (HW), head height (HH), the distance from the coronoid process of the mandible to snout tip (i.e. snout length, CT), and posterior surface of quadrate to snout tip (QT); and the ornamented measurements include casque head length (CHL), casque head height (CHH), and casque height (CH). The mass of each chameleon was also measured using a Pesola® micro-line spring scale (model 93010).

**Table 1:** Summary of morphological and bite performance data for male (M) and female (F) dwarf chameleons used in this study, grouped by phenotypic form.

	*B. melanocephalum*		Type A		*B. thamnobates*		Type B	
	M	F	M	F	M	F	M	F
*Morphology*								
*N*	*25*	*16*	*19*	*23*	*20*	*25*	*15*	*12*
SVL (mm)	49.14	57.47	48.23	45.34	60.00	66.42	68.97	77.49
	(0.88)	(0.95)	(1.68)	(1.37)	(3.27)	(3.32)	(1.20)	(1.98)
Non-ornamented								
LJL (mm)	11.55	11.05	13.27	14.29	14.39	13.56	11.06	11.65
	(0.84)	(0.86)	(3.15)	(2.29)	(3.19)	(3.06)	(2.27)	(2.56)
HL (mm)	11.68	11.23	13.08	13.96	14.47	14.17	11.12	12.72
	(0.72)	(0.85)	(2.41)	(2.18)	(2.56)	(2.76)	(1.89)	(2.60)
HH (mm)	6.98	6.89	8.44	9.08	9.00	8.73	6.81	7.42
	(0.67)	(0.47)	(2.24)	(1.65)	(1.96)	(2.10)	(1.41)	(1.45)
HW (mm)	7.54	7.33	9.28	9.87	10.41	9.84	7.51	7.87
	(0.48)	(0.59)	(2.59)	(2.10)	(2.50)	(2.60)	(1.19)	(1.71)
CT (mm)	9.05	8.82	10.22	10.94	10.84	10.57	8.48	9.07
	(0.67)	(0.80)	(2.29)	(1.71)	(2.32)	(2.41)	(1.44)	(1.64)
QT (mm)	10.29	9.79	11.82	12.50	13.08	12.18	9.51	10.36
	(0.68)	(0.87)	(3.00)	(2.05)	(2.93)	(3.00)	(1.86)	(2.15)
Ornamented								
CH (mm)	4.66	4.44	7.04	7.23	7.85	7.49	5.12	5.48
	(0.84)	(0.70)	(2.86)	(1.84)	(2.42)	(2.42)	(1.41)	(2.05)
CHL (mm)	16.75	16.18	19.90	21.46	22.28	21.25	15.75	17.44
	(1.12)	(1.20)	(4.79)	(3.90)	(4.83)	(5.12)	(3.17)	(3.94)
CHH (mm)	9.85	9.57	13.29	14.41	14.69	14.53	10.11	11.25
	(1.29)	(1.04)	(4.68)	(3.03)	(4.09)	(4.23)	(2.45)	(3.29)
*Performance*								
*N*	*23*	*15*	*19*	*20*	*20*	*25*	*13*	*12*
Bite force (N)	10.37	13.88	11.77	9.05	23.57	24.74	30.4	34.25
	(3.11)	(4.11)	(5.55)	(5.23)	(17.89)	(16.35)	(7.58)	(13.04)

Standard deviation shown in brackets. LJL, lower jaw length; HL, head length; HH, head height; HW, head width; CT, coronoid process of mandible to snout tip; QT, posterior surface of quadrate to snout tip; CH, casque height; CHL, casque head length; CHH, casque head height.

### Bite Force

Chameleons were allowed to thermoregulate in a sun/shade setting to obtain their preferred body temperature (between 28–32°C [44]). *In vivo* bite force was then measured in Newtons (N) at ambient temperature using an isometric force transducer (Kistler type 9203, ±500 N) connected to a bite plate and a Kistler charge amplifier (type 5995A, Kistler Inc., Winterthur, Switzerland) [3], [4]. The bite plate was then placed between the jaws of the chameleon, which typically resulted in the chameleon biting down on the plate repeatedly. When necessary, chameleons were induced to bite by gently tapping the sides of their jaws. Five independent measures were recorded per chameleon and the highest value retained for analysis.

### Statistical Analyses

All analyses were carried out using SPSS version 17.0 [45]. All data were log_10_ transformed prior to analysis to fulfill assumptions of normality and homoscedascity. To separate differences in shape and performance from differences in body size, all data were size-corrected against log_10_SVL and the unstandardized residuals saved for use in subsequent analyses. Although studies have shown that the head can develop at a different rate than overall body size (e.g., [46], [47]), this was not found to be the case for these chameleons. After applying the methods of Braña [46] and McCoy and colleagues [48] across all phenotypic forms and sexes, all morphometric variables were found to share a common growth axis and follow similar trajectories, and SVL was recognized as having the highest principle component loading validating its use as a suitable covariate for all measurements.

Although a previous study showed significant differences in head morphology between the four phenotypic forms and sexes in this study [33], a multivariate analysis of covariance (MANCOVA) using a general linear model (GLM), and a principle component analysis (PCA) were conducted to verify those results on this dataset. The full GLM model specified SEX and FORM as fixed factors, SEX x FORM as the interaction, log_10_SVLas the covariate, and all log_10_-tranformed head variables as the dependent variables. The unstandardized residuals for the nine head variables were then entered into a PCA and the principle component (PC) scores were saved so that the magnitude and direction of the eigenvector describing the differences between forms could be illustrated. Only PCs with eigenvalues larger than one were extracted, and the varimax rotation was used to minimize the number of variables with high loadings on each factor. Variables with communality values less than 0.5 were omitted from the analysis, as low values indicate those variables are uninformative [49]. The saved PC scores were then entered as the dependent variables in analyses if variance (ANOVAs), with FORM as the fixed factor to assess more fine-scale differences in head morphology between forms. Bonferroni post-hoc tests were run to determine which forms differed for each principal component. Next, additional ANOVAs were conducted on both absolute (log_10_-transformed) and relative (size-corrected) bite force to test for differences in performance between forms. All *P*-values were subjected to Holm’s sequential Bonferroni correction.

Because the morphological variables found to be most relevant to bite performance differ between species (e.g., [5], [13], [18], [50]), multiple regression models were carried out on size-corrected variables to explore which ones best explained the variation in bite force within each form. Akaike’s information criterion (AIC) was calculated using the residual sum of squares from each model, and the difference between the lowest AIC and all others (Δ_i_) was determined. Akaike’s weights (*wi*) were then calculated for each model, with the one exhibiting the highest *wi* acknowledged as the best fitting model [51].

## Results

Morphological and performance data were gathered from 155 dwarf chameleons within the *B. melanocephalum-B. thamnobates* species complex (Table 1). A MANCOVA revealed differences in head morphology between the four phenotypic forms (Wilks’ λ = 0.363, *F*_3,36_ = 4.890, *P<*0.001) and sexes (Wilks’ λ = 0.859, *F*_1,9_ = 2.745, *P = *0.005), with the PCA and subsequent ANOVA indicating that *B. melanocephalum* had proportionally the smallest head in both sexes, *B. thamnobates* the biggest, and Types A and B being intermediate in head size, confirming that this subset of data shows the same pattern as the previous study [33].

**Figure 4:**
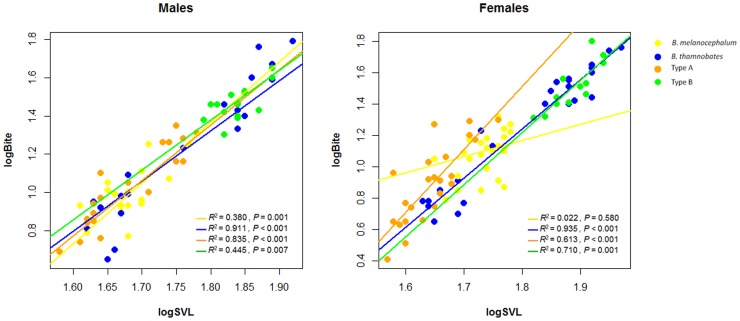
Regression plots illustrating the correlation between SVL and bite force within the *B. melanocephalum-B. thamnobates* species complex.

**Figure 5:**
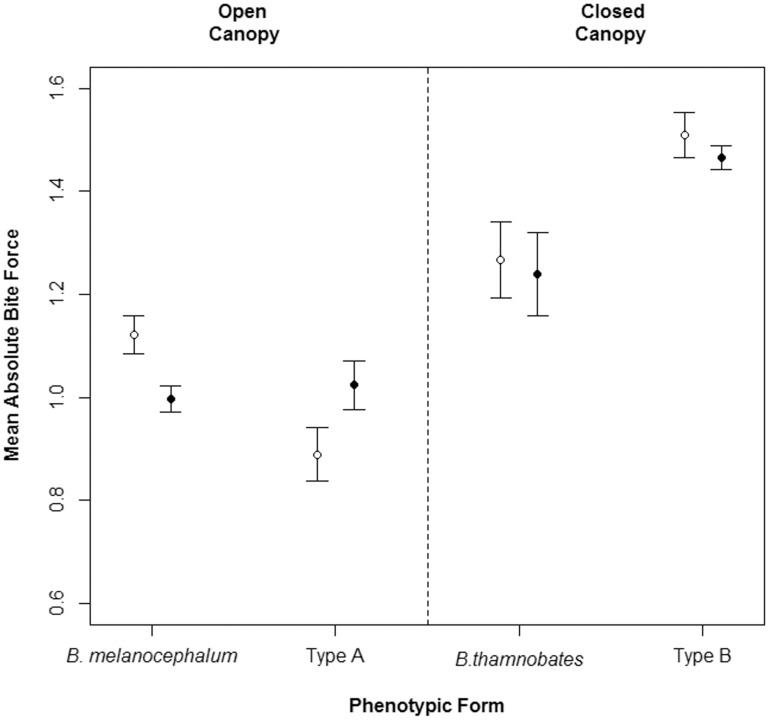
Error plots depicting mean absolute bite force for the five phenotypic forms. Absolute force equates to log_10_-transformed bite force. Solid circles represent males; empty circles, females.

**Figure 6:**
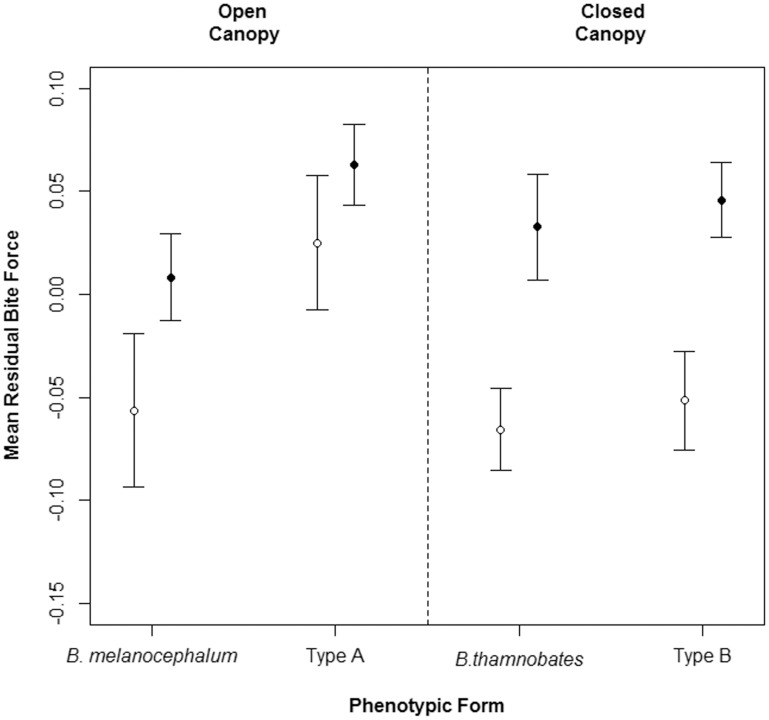
Error plots depicting mean relative bite force for the five phenotypic forms. Relative forces represent the residual values from regressing log_10_Bite Force against log_10_SVL. Solid circles represent males; empty circles, females.

Bite force was found to correlate positively with body size (SVL) in all phenotypic forms and sexes (Fig. 4). A comparison of bite performance between the sexes revealed different patterns in absolute and relative forces (Table 2). Females tended to have a stronger absolute bite force than males (Table 1), with the most pronounced difference detected in *B. melanocephalum* (*F* = 8.283, *P = *0.006; see Fig. 5). However, once bite force was corrected for body size, *B. thamnobates* and Type B males were found to bite proportionally harder than females (*B. thamnobates*: *F* = 9.437, *P = *0.004; Type B: *F* = 10.770, *P = *0.003; see Fig. 6). The open-canopy habitat forms showed no sexual variation in bite performance (*B. melanocephalum*: *F* = 2.660, *P = *0.111; Type A: *F* = 0.870, *P = *0.357).

**Table 2:** MANOVA results examining bite force differences between sexes within each of the four phenotypic forms.

	*B. melanocephalum*		*B. thamnobates*		Type A		Type B	
	*F*	*P*	*F*	*P*	*F*	*P*	*F*	*P*
Absolute Bite Force*	8.283	0.006	0.059	0.809	3.498	0.069	0.852	0.365
Relative Bite Force†	2.660	0.111	9.437	0.004	0.879	0.357	10.770	0.003

* Based on log_10_-transformed data.

† Based on residual data of log_10_Bite Force against log_10_SVL.

When examining bite force between forms, differences were only found for absolute (Males: *F*_3,78_ = 19.431, *P<*0.0001; Females: *F*_3,75_ = 13.716, *P*<0.0001) and not relative bite forces (Males: *F*_3,78_ = 1.437, *P = *0.229; Females: *F*_3,75_ = 1.575, *P = *0.189). Similar patterns were detected for both sexes, with the four phenotypic forms fitting into one of two strength categories: weak (*B. melanocephalum,* Type A) or strong (*B. thamnobates* and Type B) (Fig. 5). In males, Type B further differentiated from *B. thamnobates* by possessing a significantly stronger bite.

Model selection using linear regression to find the morphological variables that best explain bite force found different correlations between the four phenotypic forms and sexes (Table 3). *Bradypodion thamnobates* was the sole form whose performance could only be explained by a single model (Table S1), and this model was the same for both sexes (HH+CT). For the other forms, several candidate models displayed significant correlations to bite performance (Table S1). Apart from *B. thamnobates,* different parts of the casque were identified as contributing to bite force in males; however, the contribution was only significant for *B. melanocephalum* (CHL) and Type B (CH, CHH). In comparison, non-ornamented features (HH, HL, CT, QT) explained bite force in females (Table 3).

**Table 3:** Morphological models found to best reflect bite force within each phenotypic form and sex.

	Males				Females			
Phenotypic Form	Model	*ß*	*R^2^*	*P*	Model	*ß*	*R^2^*	*P*
*B. melanocephalum*	CHL	0.625	0.391	0.001	QT	−0.587	0.345	0.017
*B. thamnobates*	CT	1.335	0.334	0.031	CT	2.122	0.247	0.044
	HH	−1.421			HH	−2.292		
Type A	CH	−1.607	0.245	0.105	HH	2.045	0.362	0.033
	LJL	1.627			HL	−0.17		
					QT	−1.737		
Type B	CH	0.758	0.735	0.031	CT	3.044	0.594	0.017
	CHH	−1.218			QT	−3.049		
	HH	−0.988						
	LJL	0.78						
	CT	0.572						
Type C	CH	−0.219	0.048	0.634	CHH	0.901	0.812	0.001

All variables were size-corrected prior to analysis. *ß*, Beta coefficient; *R* ^2^, coefficient of determination; *P*, significance value; CHL, casque head length, CHH, casque head height; CH, casque height; HL, head length; HW, head width; HH, head height; LJL, lower jaw length; CT, coronoid process of mandible to snout tip; QT, posterior surface of quadrate to snout tip.

## Discussion

Head morphology and bite performance within the *B. melanocephalum-B. thamnobates* species complex is influenced by varying degrees of natural and sexual selection, and the intensity of each appears to depend, at least partly, on the structure of the habitat. For all forms, bite force was found to correlate to overall body size, with the larger, closed-canopy forms possessing a stronger bite, as predicted under natural selection. Moreover, the degree of sexual dimorphism in head shape resulted in comparable levels of dimorphism in bite performance, with closed-canopy males biting proportionally harder than females, as predicted under sexual selection, and no dimorphism in bite performance within the open-canopy forms, possibly due to natural selection curbing sexual dimorphism for increased crypsis.

The influence of selective forces on performance is typically assessed through an examination of the proportional (size-corrected) differences between groups because morphological traits, and their associated performance, typically scale with an organism’s overall body size. Consequently, differences in trait values among individuals within populations, and between populations and species, will often arise simply because individuals or populations differ in body size. With this in mind, the lack of proportional differences in bite force between phenotypic forms might suggest that natural selection is weak or not acting upon this performance measure, possibly indicating that their differential head morphologies may be a consequence of some other factor, such as founder effects. However, the absolute differences detected between open and closed-canopy forms may be of significance considering, for many animals, body size is highly heritable [52] and has been shown to be influenced by habitat use (e.g., [53], [54]). Each form approaches different body sizes [33], so the detected differences in absolute bite force are likely indicative of ecological differences between them, such as differences in diet (e.g., [12], [55]) or how they conduct their social interactions.

The snout length (CT) was the common variable found to explain bite force amongst both sexes of *B. thamnobates* and Type B – in absolute terms, the two strongest forms. The muscles attaching to the coronoid (see [56] for details) aid in bite force generation. Bite force has been associated with prey size and hardness in lizards, with animals possessing greater bite forces capable of consuming larger and/or harder prey (e.g., [12], [20], [57]). If similar correlations exist here, then these results suggest that *B. thamnobates* and Type B are likely to consume larger and/or harder prey items than *B. melanocephalum* and Type A.

Absolute bite force might also reveal something about the social system in place within each habitat. In closed-canopy habitats, larger body sizes are advantageous because they provide an honest signal of bite force, enabling chameleons to display their potential threat from farther distances through the use of their ornamentation and, if necessary, engage in combat (see [37], [58]). Chameleons in the open-canopy habitat, however, have experienced a reduction in their secondary sexual characteristics, suggesting they might be better at communicating in close proximity [5]. The casque of *B. melanocephalum* and Type A males was found to contribute to bite performance; therefore, despite its reduced size, it may be effective enough to ward off unwanted encounters at close range.

Much like between forms, absolute differences in bite performance were also detected between the sexes. The general trend showed that females bite harder than males, because they are on average, larger in body size. Even though this relationship was only significant for *B. melanocephalum*, it is possibly present within other forms, yet could not be detected due to the reduced power (*ß* <0.2) brought on by limited sample sizes. Accordingly, the greater absolute bite forces of females may reduce niche overlap [59] as has been suggested for other lizards [4], [9], [12], [60]. For these chameleons, the bite of females was dictated by non-ornamented features, namely QT which, along with CT represents the out-lever for jaw closing. Due to the high energy demands of reproduction, females often need to consume more and/or different prey items than males [61]. Considering that insect abundance and diversity can vary in vertical (canopy versus understory) and horizontal (between habitats) stratification [62]–[70], and females within the *B. melanocephalum-B. thamnobates* species complex have been found to perch lower and occupy more open-canopy habitats than males for all forms [33], the observed differences in bite performance between the sexes may allow for differences in dietary exploitation. However, a thorough dietary analysis needs to be undertaken to test this hypothesis.

The stronger bite of females may also provide them with an advantage during female-male interactions. In female dwarf chameleons, the need to mate after each litter is reduced because they have relatively long gestation periods for their body size (∼ three months) and are able to store sperm, which enables them to have asynchronous reproduction [29], [30], [38]. Consequently, 40–80% of females are gravid at a given time [38]. Moreover, female dwarf chameleons do not change colour to illustrate their receptive or gravid state [38]; therefore, the chances of males encountering a receptive female are rare. Consequently, males use courtship displays to assess a female’s willingness to mate, with females often responding with aggressive rejection behaviours [29], [30], [37], [38], including biting [37]. As a result, males tend to court smaller females, which are less able to dominate or inflict injury [37]. Considering that our study has shown that large females possess a stronger bite than small females, the aggressive behaviour of females is potentially an honest signal of their ability to ward off unwanted encounters.

In addition to sexual dimorphism in absolute bite force, relative differences were also detected with closed-canopy males biting harder than females of the same size. A likely explanation is that closed-canopy habitats allow for increased competition between males for access to females, as was found with increased colour change within these habitats [3], resulting in a greater investment in the jaw muscle in males, which is also reflected in their proportionally higher and longer heads. Indeed, snout length and head height were found to best explain male bite performance, possibly by increasing the available space for jaw adductor muscles, resulting in a higher physiological cross-section and hence bite force [4], [50], [71]. This is particularly relevant because altercations between males can be aggressive and often involve biting [29], [36]. Within Type B males, the casque (CH, CHH) was also found to contribute to bite performance, and is almost certainly used as an honest visual signal, notifying other males of the potential cost of fighting. Even though the casque did not explain bite force in *B. thamnobates*, it still appears to be an honest signal of bite performance, as larger bodied males possess larger casques and have a correspondingly harder bite.

Within the open-canopy forms (*B. melanocephalum* and Type A), little to no sexual dimorphism in head morphology was uncovered, which resulted in a lack of dimorphism in bite performance. The comparable bite forces between the sexes suggests that within more open-canopy habitats there is either reduced direct competition between males for access to females or the need for increased crypsis is so strong it outweighs intrasexual selection. While there is no evidence to support the former, the trade-off between crypsis and communication/signalling ability in dwarf chameleons has been studied extensively [3], [5], [72], [73]. For example, the spectral properties of chameleon signals varies predictably with habitat structure, with the display colours of open-canopy chameleons having lower UV reflectance than that of closed-canopy chameleons [73]. High UV reflectance has been found to increase an animal’s detectability [74]; and, although, the low reflectance of open-canopy chameleons decreases their detectability to conspecifics, it is also thought to protect them from UV-sensitive avian predators [3]. Accordingly, natural selection is likely to be the predominant force in open-canopy habitats, inhibiting the development of conspicuous secondary sexual characteristics and, ultimately, enforcing their overall diminutive body size and constraining performance. However, the casque was found to contribute to bite force in the open-canopy habitat forms (*B. melanocephalum*: CHL; Type A: CH) and likely acts as an honest signal of performance, indicating that sexual selection might also be influencing performance in these chameleons. In fact, both selective forces are certainly operating simultaneously, but to varying degrees in each habitat.

Similar habitat-specific sexual differences have helped explain the ecomorphological diversity produced by the adaptive radiations of West Indian *Anolis* lizards [21], [24], [59], [75]. In general, anoles in low-visibility microhabitats, such as the tree crown which has dense branches and leaves, tend to have low dimorphism; whereas those in high-visibility microhabitats, such as the tree trunk or open ground, have high dimorphism [21], [75]. This relationship is similar to that found with the KZN dwarf chameleons given that the microhabitats of the open-canopy forms were actually found to have a higher density of perches and, hence, are more likely to have low-visibility, and vice versa in the closed-canopy habitats [33]. The overall extent of sexual variation in anoles can be so great, in fact, that it can exceed interspecific variation [24]. Consequently, overlooking sexual dimorphism could underestimate the adaptive component of an evolutionary radiation [24]. In light of this, sexual dimorphism should be deemed yet another ecomorphological trait used to assess divergence within a radiation or species complex. Accordingly, this study, coupled with the functional differences in forefoot grip strength already detected between open and closed-canopy forms in this species complex [35], proves that these five phenotypic forms have adapted morphologically to their different environments.

## Supporting Information

Table S1 **Regression models exploring the best morphological correlate of bite force for each of the five phenotypic forms of the** ***B. melanocephalum*** **-** ***B. thamnobates*** **species complex.**
(DOCX)
