# Supplementary material for: Sexual Dimorphism in Bite Performance Drives Morphological Variation in Chameleons
Source: PLoS One. 2014 Jan 27;9(1):e86846. doi: 10.1371/journal.pone.0086846 (PMC3903609; doi:10.1371/journal.pone.0086846)
Supplement: Table S1 — Regression models exploring the best morphological correlate of bite force for each of the five phenotypic forms of the B. melanocephalum - B. thamnobates species complex. (DOCX) [file pone.0086846.s001.docx]

Table S1. Regression models exploring the best morphological correlate of bite force for each of the five phenotypic forms of the *B. melanocephalum*-*B. thamnobates* species complex.

|  | Males | | | Females | | |
| --- | --- | --- | --- | --- | --- | --- |
| Phenotypic form | Model | AIC | *wi* | Model | AIC | *wi* |
| *B. melanocephalum* | HL | -110.395 | 0.010 | HL | -56.730 | 0.022 |
|  | HW | -116.242 | 0.182* | HW | -60.216 | 0.123* |
|  | HH | -111.388 | 0.016* | HH | -52.630 | 0.003 |
|  | LJL | -113.507 | 0.046* | LJL | -56.882 | 0.023 |
|  | CT | -110.287 | 0.009* | CT | -59.118 | 0.071 |
|  | QT | -115.697 | 0.139* | QT | -61.539 | 0.239* |
|  | CH | -109.546 | 0.006 | CH | -53.987 | 0.005 |
|  | CHH | -107.544 | 0.002 | CHH | -56.188 | 0.016 |
|  | **CHL** | **-117.809** | **0.399*** | CHL | -55.480 | 0.012 |
|  | HW+CT+QT | -114.557 | 0.047* | **HL+HW+QT+CH+ CHL** | **-68.136** | **0.269*** |
|  | HW+QT | -115.084 | 0.084* | HW+CT+QT | -59.932 | 0.042 |
|  | HW+CH | -114.380 | 0.059* | HW+QT | -61.611 | 0.175* |
| *B. thamnobates* | HL | -80.437 | 0.046 | HL | -109.546 | 0.058 |
|  | HW | -80.437 | 0.046 | HW | -110.18 | 0.079 |
|  | HH | -80.981 | 0.061 | HH | -109.967 | 0.071 |
|  | LJL | -80.208 | 0.041 | LJL | -109.755 | 0.064 |
|  | CT | -80.208 | 0.041 | CT | -109.132 | 0.047 |
|  | QT | -80.437 | 0.046 | QT | -109.441 | 0.055 |
|  | CH | -80.981 | 0.061 | CH | -109.234 | 0.049 |
|  | CHH | -80.513 | 0.048 | CHH | -109.755 | 0.064 |
|  | CHL | -80.513 | 0.048 | CHL | -109.546 | 0.058 |
|  | **HH+CT** | **-85.950** | **0.562*** | **HH+CT** | **-113.697** | **0.378*** |
|  |  |  |  | HW+CT+QT | -111.163 | 0.078 |
| Type A | HL | -86.751 | 0.058 | HL | -80.012 | 0.048 |
|  | HW | -86.751 | 0.058 | HW | -79.805 | 0.043 |
|  | HH | -86.613 | 0.054 | HH | -80.990 | 0.078 |
|  | LJL | -86.751 | 0.058 | LJL | -79.518 | 0.038 |
|  | CT | -86.890 | 0.062 | CT | -80.053 | 0.049 |
|  | QT | -86.751 | 0.058 | QT | -79.518 | 0.038 |
|  | CH | -86.751 | 0.058 | CH | -81.121 | 0.084 |
|  | CHH | -86.613 | 0.054 | CHH | -80.903 | 0.075 |
|  | CHL | -86.751 | 0.058 | CHL | -80.860 | 0.074 |
|  | **LJL+CH** | **-89.654** | **0.189** | **HL+HH+QT** | **-85.225** | **0.370*** |
|  | HH+LJL+ CH | -89.171 | 0.094 | HW+HH+CT+QT+CHH | -85.085 | 0.103* |
|  | LJL+QT+CH | -89.774 | 0.127 |  |  |  |
|  | LJL +CT+QT+CH | -89.946 | 0.070 |  |  |  |
| Type B | HL | -74.335 | 0.060 | HL | -52.580 | 0.016 |
|  | HW | -73.431 | 0.039 | HW | -52.580 | 0.016 |
|  | HH | -73.651 | 0.043 | HH | -52.580 | 0.016 |
|  | LJL | -72.582 | 0.025 | LJL | -52.580 | 0.016 |
|  | CT | -72.378 | 0.023 | CT | -52.724 | 0.017 |
|  | QT | -73.214 | 0.035 | QT | -52.724 | 0.017 |
|  | CH | -74.103 | 0.054 | CH | -52.724 | 0.017 |
|  | CHH | -72.582 | 0.025 | CHH | -53.017 | 0.020 |
|  | CHL | -72.378 | 0.023 | CHL | -52.580 | 0.016 |
|  | HH+HW | -76.677 | 0.134 | **CT+QT** | **-60.588** | **0.508** |
|  | LJL+CT+CH | -70.335 | 0.003 | HW+CT+QT | -58.588 | 0.071 |
|  | HH+HL+ QT | -76.011 | 0.050 | CT+QT+CH | -60.955 | 0.233* |
|  | HL+HW+CHH | -76.011 | 0.059 | HL+QT+CT+CH+CHL | -65.985 | 0.040* |
|  | HH+HW+LJL+CT+CH+CHH | -88.085 | 0.171* |  |  |  |
|  | **HH+LJL+ CT+CH+CHH** | **-83.990** | **0.210*** |  |  |  |
|  | HL+HW+HH +LJL+CT+CH+CHH+CHL | -102.275 | 0.046* |  |  |  |
| Type C | HL | -17.263 | 0.104 | HL | -21.113 | 0.002 |
|  | HW | -17.414 | 0.112 | HW | -22.026 | 0.003 |
|  | HH | -17.214 | 0.101 | HH | -21.167 | 0.002 |
|  | LJL | -17.288 | 0.105 | LJL | -22.943 | 0.005 |
|  | CT | -17.288 | 0.105 | CT | -22.026 | 0.003 |
|  | QT | -17.239 | 0.102 | QT | -21.946 | 0.003 |
|  | **CH** | **-17.517** | **0.118** | CH | -27.591 | 0.051* |
| Type C | CHH | -17.239 | 0.102 | **CHH** | **-33.300** | **0.879*** |
| continued | CHL | -17.214 | 0.101 | CHL | -23.196 | 0.006 |
|  | CH+CHL | -18.765 | 0.049 | HH+CH | -25.632 | 0.008 |
|  |  |  |  | CT+CH | -26.764 | 0.014* |
|  |  |  |  | HW+CH | -25.838 | 0.009 |
|  |  |  |  | CH+CHL | -26.954 | 0.015* |

AIC, Akaike’s information criterion; *wi*, Akaike’s weight; CHL, casque head length, CHH, casque head height; CH, casque height; HL, head length; HW, head width; HH, head height; LJL, lower jaw length; CT, coronoid process of mandible to snout tip; QT, posterior surface of quadrate to snout tip; *, *P* < 0.05. Text in bold highlights the best fitting model.
